# Effect of Humidity on Ionisation and Ion Chemistry in Active‐Capillary Dielectric Barrier Discharge Ionisation

**DOI:** 10.1002/rcm.70130

**Published:** 2026-06-28

**Authors:** Miroslav Polášek, Patrik Španěl, Kseniya Dryahina

**Affiliations:** ^1^ J. Heyrovský Institute of Physical Chemistry of the Czech Academy of Sciences Praha Czechia

**Keywords:** active capillary, atmospheric pressure ionisation, DBDI, humidity, SICRIT

## Abstract

**Rationale:**

Dielectric barrier discharge ionisation (DBDI) in an active‐capillary configuration (including the commercial SICRIT source) is widely used as a soft, ambient‐pressure ionisation method for vapours and gases, yet the role of water vapour in controlling reagent ion chemistry and analytical behaviour has not been mechanistically described. Thus, it is necessary to understand how carrier gas humidity governs reagent ion populations and product ion formation in a DBDI source.

**Methods:**

A SICRIT ion source was coupled to a triple‐quadrupole mass spectrometer and operated under constant discharge conditions while switching the carrier gas between humidified and nominally dry air. Ketones and short‐chain carboxylic acids were introduced at trace vapour concentrations using permeation or diffusion tubes. Reagent ion and analyte mass spectra were acquired in full‐scan mode. Selected analyte ions were subjected to collision‐induced dissociation (CID) to compare in‐source fragments with those formed from protonated molecules.

**Results:**

Under humid conditions, the reagent ion population was dominated by hydrated hydronium ions. Analytes yielded mainly protonated molecules, MH^+^ and their hydrates, with minimal fragmentation. Switching to a dry carrier gas reduced the total ion current by more than an order of magnitude and led to extensive, structure‐dependent fragmentation. Comparison with CID spectra of MH^+^ indicates that the fragments observed under dry conditions arise from the fragmentation of protonated molecules rather than from the direct dissociation of radical cations. This is explained by highly exothermic proton transfer from a dominant *m/z* 29 reagent ion (most plausibly N_2_H^+^).

**Conclusions:**

These results highlight humidity as a key operational active‐capillary DBDI parameter; varying it enables both low‐fragmentation ionisation in humid mode and more energetic fragmenting ionisation in dry mode. The humid mode facilitates reproducible quantitation, whereas the dry mode allows structural discrimination, exemplified by differentiation of hexanone isomers.

## 1 Introduction

Dielectric barrier discharge ionisation (DBDI), operated in an active‐capillary configuration [1], is becoming an increasingly popular atmospheric pressure soft ionisation technique for mass spectrometric analysis of vapourised and gaseous samples. The success in active‐capillary DBDI development started in 2012 with the presentation of a new active‐capillary plasma source (ACaPI) [2] configuration for ambient mass spectrometry and resulted in the availability of a commercial SICRIT ion source (Soft Ionisation by Chemical Reaction In Transfer) designed as an add‐on to conventional mass analysers, offering high sensitivity and minimal sample preparation [1]. The active‐capillary DBDI has been shown to be ‘softer’ than previously used techniques (e.g., APCI and DART), resulting in less fragmentation [3, 4] with average internal energy distribution over 30% lower than for other DBD‐based sources (LTP and F*μ*TP) [4]. In an active‐capillary configuration, gaseous analytes are driven towards the mass spectrometer capillary inlet through the centre of a DBD halo‐plasma, which minimises interactions between the plasma and the vaporised molecules to reduce the energy deposited during ionisation [4]. This makes active‐capillary DBDI attractive for real‐time, in situ and direct analysis across various fields, including environmental monitoring [5], analysis of explosives [6], food science [7, 8], forensic studies [9] and biomedical research [10, 11].

A typical active‐capillary DBDI ring‐to‐ring ion source features a direct flow‐through interface to a mass spectrometer, facilitating efficient sampling and rapid real‐time analyses. This source is a miniature device comprising two concentric ring‐shaped electrodes separated by a dielectric capillary. The vacuum system of the MS instrument maintains a fixed discharge gas flow rate through the ion transfer capillary. Usually, the discharge gas is the sample gas (containing analyte molecules), diluted with a carrier gas (such as air or nitrogen), sometimes with an added makeup gas (e.g., water vapour in humidified air). Plasma is generated inside the dielectric capillary by a dielectric barrier discharge powered by a high‐frequency (10–20 kHz) AC electric field, which accelerates electrons to ionise the passing discharge gas containing the analyte molecules. The DBDI produces a non‐thermal cold plasma, where the electron temperature is much higher than the gas temperature, allowing effective ionisation under near‐ambient conditions without significant gas heating.

The ionisation processes in active‐capillary DBDI are complex [1], involving a network of gas‐phase reactions, radical formation, charge transfer and collisions in both the discharge and afterglow regions [12]. Several experimental parameters, such as the applied voltage, the composition of the discharge gas and the presence of water vapour, play a crucial role in determining plasma chemistry and ion formation. The discharge voltage influences electron energy and the degree of ionisation; higher voltages generally increase electron density and excitation efficiency, which can lead to enhanced ion yields but also to increased fragmentation due to more energetic collisions. The effect of discharge voltage on ion formation in DBDI has been investigated and reported in several recent studies. Specific fragmentation, which occurred in a nitrogen atmosphere at an operating voltage of 3.4 kV, could be used to differentiate regioisomers of alkylated aromatic hydrocarbons in positive mode [13]. A similar effect of the discharge voltage on ion formation has been observed for perfluorinated alkanes in negative mode [14]. At low discharge voltages, the dominant product ion was the molecular radical anion [M]^−•^, formed predominantly via electron capture. In contrast, at higher voltages, the [M − F]^−^ ion became the major product, attributed to electron capture dissociation processes occurring under more energetic plasma conditions.

Previous work has shown that active‐capillary DBDI/SICRIT ionisation is sensitive to the composition of the discharge or makeup gas. For example, Weber et al. [15] systematically investigated the ionisation behaviour of 15 different compound classes with the SICRIT ionisation source under eight different makeup gas compositions. Compounds with comparatively high proton affinities (PAs), for example, those containing functional groups such as amino, ester, carbonyl or nonconjugated double bonds, are predominantly protonated, whereas dry nitrogen produces the highest amount of fragment ions. Air and humidified nitrogen provided the best ionisation efficiency, even if they are slightly less effective for compounds with low PA. They also offer the benefit of lower or no fragmentation, even for labile compounds.

Humidity in the discharge gas is particularly significant. The presence of water vapour affects the population of reactive oxygen and nitrogen species, modulates electron energy distribution and promotes the formation of hydrated hydronium ions H_3_O^+^(H_2_O)_n_, which act as efficient proton donors in soft ionisation processes. Typically, active‐capillary DBDI sources operate effectively using only the moisture naturally present in the ambient atmosphere, as even small amounts of water vapour are sufficient to sustain hydronium‐based ion chemistry. However, if there is an aim to avoid impurities from the environment and clean dry gas is used as a carrier gas in a closed system, additional humidification is necessary. According to the SICRIT user manual, ‘to improve the ionisation efficiency, a gas humidifier should be installed in closed sampling systems’, underlining the beneficial role of controlled humidity in enhancing ionisation efficiency [16].

The presence of water vapour thus plays a key role in the soft ionisation nature of the practical DBDI ion sources. The previous work by Gyr et al. [17] showed that ACaPI's discharge gas composition and humidity (air, dry and humidified nitrogen or carbon dioxide) affect the reagent ions generated and downstream chemistry. This is because even small concentrations of water molecules react with the primary ions formed in the discharge to form protonated H_3_O^+^ reagent ions and their hydrates, which then react by proton transfer with analyte molecules M to form product ions MH^+^ without undergoing significant fragmentation [12]. This mechanism provides a gentle, low‐energy ionisation pathway that preserves the molecular ion, consistent with the typical description of the SICRIT ion source as a very soft ionisation mechanism that enables analyte ionisation with minimal fragmentation.

Evidence from previously established ambient plasma‐based sources supports the practical importance of humidity control. In direct analysis in real time (DART), seasonal variation in mass spectra of the unstable explosive hexamethylene triperoxide diamine (HMTD) motivated a controlled study showing that changes in humidity can strongly affect relative ion abundances and fragmentation patterns at approximately constant temperature [18]. These findings highlight a general risk for ambient plasma ionisation methods: Uncontrolled water vapour can alter reagent ion populations and shift product ion distributions.

Studies of dielectric barrier discharges at lower pressures further emphasise how sensitively DBD ionisation responds to gas composition and water content. Sugiyama et al. introduced a low‐pressure DBD ion source positioned in a vacuum, reporting predominantly protonated molecules with only minor fragmentation and high sensitivity for selected vapours [19]. More recently, Ye et al. [20] combined optical emission spectroscopy and mass spectrometry to characterise a ring–ring low‐pressure air DBD, demonstrating pressure‐dependent behaviour of excited species and providing evidence for Penning ionisation capability in the discharge. Although these studies operate at pressures lower than in the active‐capillary DBDI under ambient conditions, they reinforce a central point directly relevant here: Reagent ion chemistry in DBD‐based ion sources is highly sensitive to the discharge environment, including the availability of water and the resulting competition among ionisation channels.

Despite the widespread practical use of humidification in DBDI and its clear mechanistic rationale, a quantitative, systematic understanding of how humidity affects reagent ion populations and analyte ionisation pathways in active‐capillary DBDI remains limited. It is not well understood how controlled adjustments in carrier gas humidity influence reagent ion composition, hydration, and product ion distribution and fragmentation. This has implications for analytical sensitivity and quantitation robustness.

In this study, we therefore systematically investigate how varied humidity in the carrier gas supplied to an active‐capillary SICRIT DBDI source affects ionisation chemistry. We focus on changes in reagent ion composition and hydration and on shifts in product ion distributions and fragmentation under otherwise fixed operating conditions. Additionally, we examine the analytical implications for sensitivity and the potential for differentiating selected structural isomers under dry carrier gas conditions. This provides both mechanistic insight and practical guidance for tuning active‐capillary DBDI performance via humidity control.

## 2 Experimental Section

### 2.1 DBDI‐MS Instrument

All experimental work for this study was carried out using the SICRIT ion source (Plasmion GmbH, Germany) coupled to a TSQ Fortis triple‐quadrupole mass spectrometer (Thermo Scientific). This active‐capillary DBDI ion source with ring‐to‐ring configuration (see Figure S12) was operated in positive mode with an amplitude of 1.8 kV and a frequency of 15 kHz. The sheath and sweep gas flow rates of the TSQ were set to zero. The ion transfer tube temperature was 300°C; the ion transfer optics parameters were set as follows: ITT potential 80 V, skimmer potential 10 V and tube lens potential 100 V.

**FIGURE 1:**
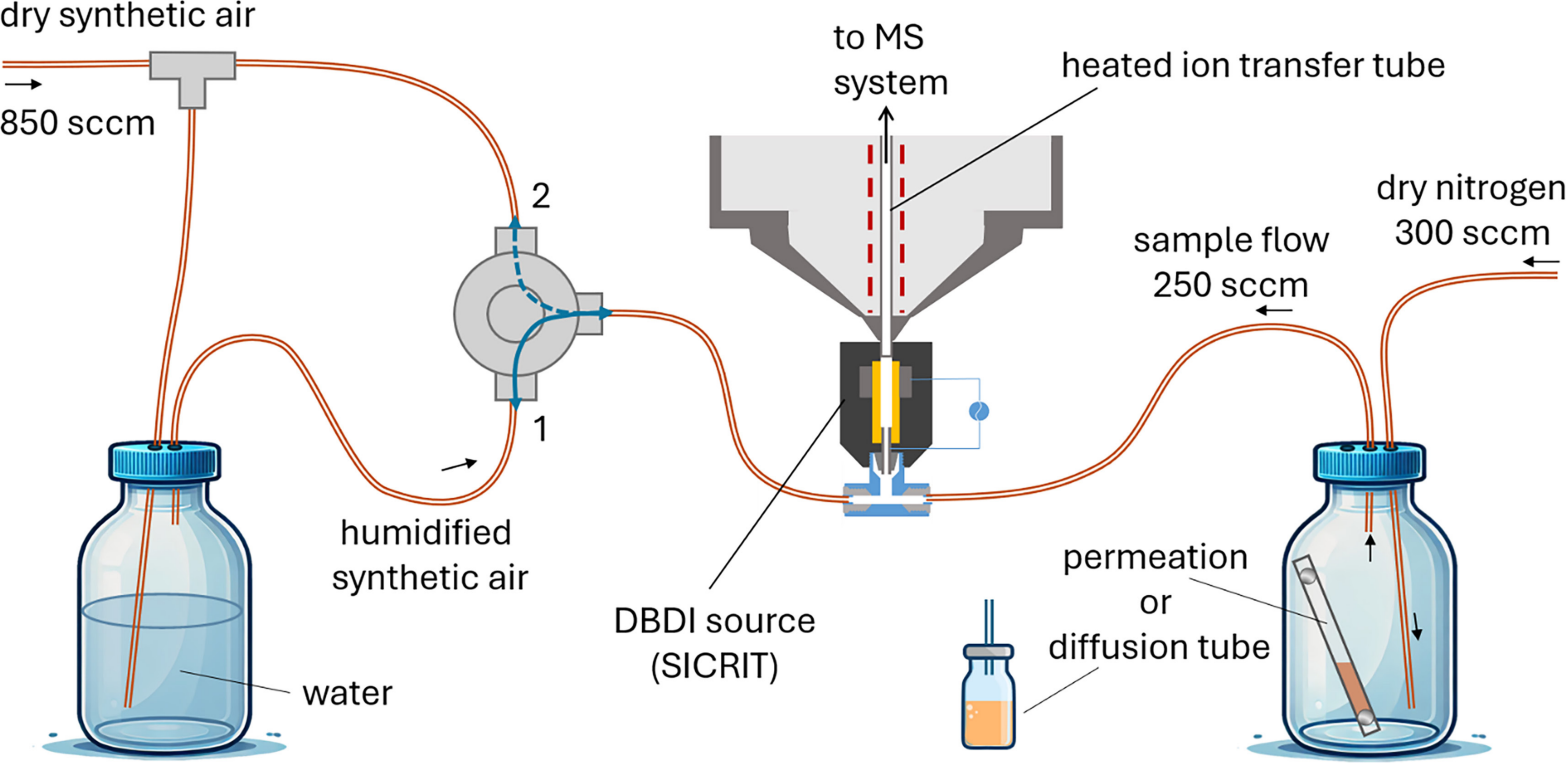
Experimental set‐up.

The inlet of the SICRIT source was connected to a Swagelok union tee (see Figure 1). Additional clean air, used as the carrier gas, was supplied through one port, with the flow rate regulated by a flow controller and set to 850 sccm. This carrier gas composition was selected to reproduce conditions in a typical ambient atmosphere operation of the ion source while allowing controlled humidity adjustment. A gaseous sample was introduced through the opposite port at an approximate flow rate of 250 sccm. This additional flow compensates for the difference between the gas flow drawn by the MS vacuum system and the lower carrier gas flow, resulting in a slight underpressure inside the ion source chamber. This ensures a stable flow through the sampling capillary. Under these conditions, the final pressure inside the source was slightly below atmospheric pressure (approximately 970 mbar). Nitrogen was used as the sample gas because its composition is closest to ambient air while minimising the influence of trace impurities naturally present in laboratory air and only minimally affecting the overall composition of the discharge gas. Switching between dry and humidified carrier gas was achieved using a switching valve system and a 500‐mL bottle containing 200 mL of water, connected to one of its lines. In the humid configuration, the valve was turned to position ‘1’, allowing dry, clean air to bubble through the water before reaching the ion source. In the dry configuration, the valve was switched to position ‘2’.

### 2.2 Chemicals and Sample Preparation

All chemicals used in this study were obtained from Sigma‐Aldrich. They were declared by the supplier to be at least 98% pure and were used in the experiments without further purification. Synthetic air 5.0 and nitrogen 5.0 (both from Messer) were used as the carrier and sample gases, respectively. Milli‐Q water was used to humidify the carrier gas. Argon 5.0 (Messer) was used as a collisional gas for CID.

*Permeation tubes* were used to generate a steady flow of sample gas containing low concentrations of specific VOCs. A pure liquid analyte (1 mL) was sealed inside an 8‐cm‐long Teflon tube (O.D. 1/4 in., I.D. 0.228 in.) using stainless steel balls. After preparation, the tubes were stored for at least 1 week in a holder inside a fume hood at room temperature to allow evaporation of any analyte residues remaining on the outside of the balls and to establish stable permeation through the tube walls. For the experiment, individual permeation tubes were placed inside a 500‐mL bottle, which was continuously flushed with a 300‐sccm flow of nitrogen. This method, based on diffusion through the tube wall, enables the generation of gas standards at trace concentrations. This approach was applied to all ketones examined in this study.

*Diffusion tubes* allowed the preparation of gas standard mixtures at higher concentrations than permeation tubes. Each diffusion tube included a reservoir (a 1‐mL glass vial holding 10 μL of a pure liquid compound) and a vial cap with a capillary tube, which acted as the diffusion path. The compound evaporated from the reservoir and diffused through the capillary into a flowing nitrogen sampling gas. They were then used similarly to permeation tubes: placed inside a 500‐mL bottle with a constant nitrogen flow. This method was employed for all acids involved in this study.

### 2.3 Experimental Protocol and Data Processing

**FIGURE 2:**
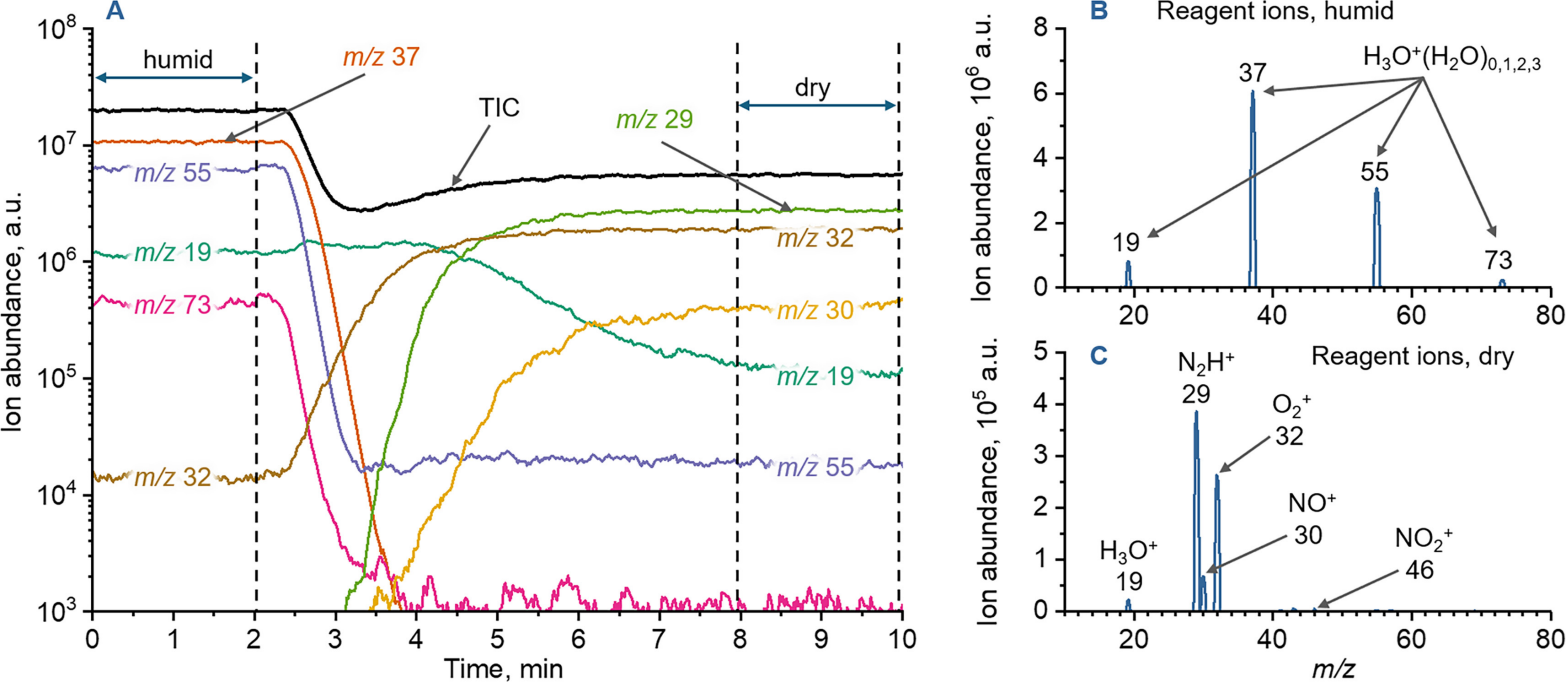
Time profile (A) and spectra of reagent ions under humidified (B) and dry (C) carrier gas conditions.

Data were collected using the TSQ Fortis 3.2 Tune application. Initially, the switching valve was set to position ‘1’ for a humid configuration and allowed to equilibrate for at least 8 min. Data collection started after this equilibration period. Two minutes after beginning data collection, the valve was switched to position ‘2’. Data were recorded until equilibrium was reestablished and continued for at least two more minutes to achieve a dry configuration. The mass spectra were obtained by scanning Q1 in positive mode over a mass range of *m/z* 10–300 at a scan rate of 250 Da/s, with a 0.7 FWHM Q1 resolution and without source fragmentation. The spectra for humid and dry configurations were obtained using Xcalibur software by averaging over the first 2 min and the last 2 min of the chromatogram, respectively (see Figure 2).

Collision‐induced dissociation (CID) mass spectra were recorded for selected precursor ions, mostly protonated analyte molecules, under humidified carrier gas conditions. This was performed in the ‘Product Ion Scan’ mode. The collision energy was set to 20 V, and the gas pressure was adjusted to produce intense signals from the fragment ions while still maintaining a visible signal from the precursor ion. The CID gas pressure was set to either 1.3 × 10^−3^ or 2.7 × 10^−3^ mbar for different precursor ions. The data acquisition time for the CID spectra was 3 min, and average scans were exported using Xcalibur software.

For the ion‐molecule reaction experiments with ion *m/z* 29, the argon collision gas was replaced with CO_2_ 4.5 (Messer CANGas). The CO_2_ pressure was set to 0.7 × 10^−4^ mbar and the collision energy to 0 V.

## 3 Results and Discussion

### 3.1 Reagent Ions Under Humidified and Dry Carrier Gas Conditions

We initially investigated how the reagent ion composition responds to changes in carrier gas humidity. For this purpose, an empty glass bottle, flushed with high‐purity nitrogen, was connected to the sample line to provide a blank (analyte‐free) stream. Figure 2A shows the time variation of the reagent ion signals as the carrier gas is switched from humidified to dry. Under humid carrier gas conditions, the reagent ion population observed at the MS analyser was dominated by hydrated hydronium ions (Figure 2B), with H_3_O^+^(H_2_O)_1,2_ showing the highest intensities. The elevated transfer capillary temperature promotes dehydration of hydrated ions, and this should be considered when interpreting the relative abundances of hydrates versus bare MH^+^ ions, as their proportions in the colder, high‐pressure region will differ.

Upon switching from humidified to dry carrier gas, the total ion current (TIC) decreased by more than an order of magnitude. Hydrated ions disappeared completely, leaving only a small amount of H_3_O^+^. The residual formation of protonated water ions is likely due to trace water impurities (< 5 ppmv) in the synthetic air, so an absolutely dry condition was not achieved, even when an additional moisture cartridge filter (Thermo Fisher Scientific) was inserted between the switching valve and the ion source.

Besides H_3_O^+^ and the dominant ion at *m/z* 29, O_2_^+•^ and NO^+^ reagent ions were formed along with a small amount of NO_2_^+^. Similar reagent ions were previously observed by Sugiyama et al. at low pressure in a DBDI source [19]. In their study, the relative intensities of NO^+^ and O_2_^+•^ reagent ions decreased with increasing pressure, whereas those of hydrated H_3_O^+^ increased. Above 500 Pa, hydrated hydronium ions became the dominant component among the reagent ions. This effect could result from an increased amount of water in the ion source at higher pressure. The formation of these ions was also observed in the atmospheric sampling glow‐discharge ionisation source [21]. The detailed ion chemistry leading to the formation of reagent ions in different carrier gases has been previously described in a review [1] and will not be discussed here.

The formation and composition of the most abundant reagent ion at *m/z* 29 is rather peculiar. Because a low‐resolution mass analyser was used in our set‐up, the elemental composition of this ion could not be determined experimentally. There are only four possible combinations of C, H, O and N atoms that could form an ion at *m/z* 29: HCO^+^, C_2_H_5_^+^, CH_3_N^+•^ and N_2_H^+^. The CID spectrum of this ion showed only one fragment ion at *m/z* 28. No signals were detected in the region between *m/z* 12 and 16, most likely due to the quadrupole mass analyser's low mass cut‐off. Nevertheless, C_2_H_5_^+^ and CH_3_N^+•^ compositions could be ruled out due to the absence of fragment ions formed by the loss of more than one hydrogen atom (i.e., *m/z* 27 and lower).

To determine experimentally whether the *m/z* 29 ion is HCO^+^ or N_2_H^+^, an ion‐molecule reaction with CO_2_ (in the collision cell of the triple quadrupole) was used.

The PAs of CO, N_2_ and CO_2_ are 594, 493.8 and 540.5 kJ mol^−1^, respectively [22]. This means that proton transfer from N_2_ to CO_2_ is exothermic:

(1) $$N_{2}H^{+}+{\mathrm{CO}}_{2}\to {\text{HOCO}}^{+}+N_{2}\;\text{∆}{H_{r}}^{{}^{\circ}}=-46.7\;\mathrm{kJ}\;{\mathrm{mol}}^{-1}$$

whereas the analogous proton transfer from CO to CO_2_ would be endothermic.

(2) $${\mathrm{HCO}}^{+}+{\mathrm{CO}}_{2}\to {\text{HOCO}}^{+}+\mathrm{CO}\;\text{∆}{H_{r}}^{{}^{\circ}}=+53.5\;\mathrm{kJ}\;{\mathrm{mol}}^{-1}$$

In other words, if *m/z* 29 is N_2_H^+^, a product ion at *m/z* 45 should be observed from Reaction (1) under near thermal conditions achievable on the triple quadrupole mass spectrometer used in this study, whereas HCO^+^ should be unreactive.

The obtained product ion mass spectra (Figure S13) clearly show that ions *m/z* 29 formed under dry DBDI conditions react with CO_2_, yielding a substantial number of ions at *m/z* 45, proving the presence of N_2_H^+^. However, the presence of a certain fraction of HCO^+^ cannot be excluded by this experiment. After all, the enthalpy of formation of HCO^+^ is much lower than that of N_2_H^+^ [23], and the carrier gas used for our experiments contained trace amounts of CO_2_ (≤ 0.5 ppmv) and small hydrocarbons (≤ 0.1 ppmv) as possible sources of carbon atoms.

Finally, note that even the slightest amount of humidity would quickly convert either N_2_H^+^ or HCO^+^ to H_3_O^+^ via fast proton transfer to H_2_O, so the exact nature of *m*/*z* 29 is not important in humid conditions, but it is highly relevant to explaining the fragmentation observed in dry conditions.

### 3.2 Analysis of Ketones Under Different Humidity Conditions

**FIGURE 3:**
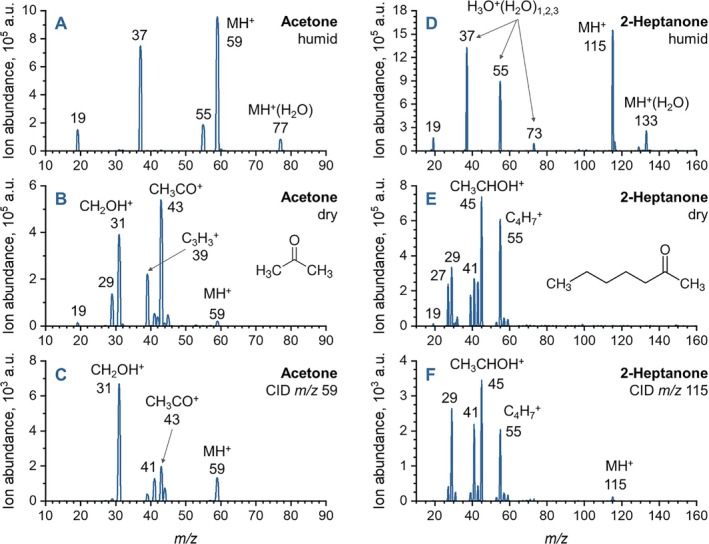
Mass spectra for acetone and 2‐heptanone: (A, D) source DBDI spectra obtained under humidified carrier gas conditions, (B, E) source DBDI spectra obtained under dry carrier gas conditions and (C, F) CID spectra (Ar, 1.3 × 10^−3^ mbar, 20 V) of protonated molecules MH^+^ obtained under humid conditions.

A similar experimental protocol to that used for reagent ion characterisation was applied to the analytes, except that the sample bottle contained permeation tubes that generated low concentrations of the selected ketones. Under humidified carrier gas conditions, the dominant product ions were protonated molecular ions MH^+^, along with a small fraction of hydrated protonated species MH^+^(H_2_O) (Figures 3A,D, S1A and S2A). At higher analyte concentrations, proton‐bound complexes 2M·H^+^ were also detected. In contrast, when the carrier gas was dry (Figures 3B,E, S1B and S2B), extensive fragmentation was observed, resulting in characteristic fragment ions for each compound.

The main product formed when acetone vapours were introduced into the DBDI source under dry conditions was an ion at *m/z* 43, corresponding to (M − CH_3_)^+^, which is commonly observed upon electron ionisation; however, this ion was also observed in CID mass spectra of protonated acetone [24] (Figure 3C). As a small amount of the protonated acetone was formed under dry DBDI conditions (Figure 3B), it can be speculated that this species is formed primarily from neutral molecules by exothermic proton transfer from N_2_H^+^ ions, as it was already studied by experiment and quantum‐chemical calculations showing that methane elimination can proceed via direct proton transfer to the methyl group [25].

An alternative mechanism involving electron transfer in the reaction of acetone molecules with O_2_^+^ is also thermochemically feasible. This electron transfer is 2.37 eV exothermic (IE(O_2_) = 12.07 eV; IE(acetone) = 9.70 eV) [22], so the nascent acetone radical cation could have enough internal energy for the fragmentation to acetyl cation (AE = 10.4 eV) [22].

The CID mass spectrum of the ion at *m/z* 43 from dry DBDI of acetone (Figure S8B) exhibits a loss of CO, producing the ion at *m/z* 15, CH_3_^+^. Another low‐abundant ion appears at *m/z* 42, corresponding to a loss of a hydrogen atom. For comparison, the CID mass spectrum of the ion at *m/z* 43 from acetic acid was recorded under dry DBDI conditions (Figure S8A, Section 3.3). In this spectrum, almost no signal was observed at *m/z* 42, leaving CO loss as the only fragmentation channel. It can be concluded from these observations that although acetic acid yields a pure acetyl cation, CH_3_CO^+^, the peak at *m/z* 43 from dry DBDI of acetone consists of acetyl cation with a certain admixture of a different isomer, probably a protonated ketene, CH_2_COH^+^, which is the second most stable isomer of the [C_2_, H_3_, O]^+^ family [26].

Another significant product was an ion at *m/z* 31, corresponding to hydroxymethyl cation, CH_2_OH^+^. This product most likely originates from the fragmentation of protonated acetone. Under dry conditions, the most abundant proton donor is N_2_H^+^ (as shown earlier in Section 3.1). The difference between the PA of N_2_ (493.8 kJ mol^−1^) and acetone (812 kJ mol^−1^) is 318.2 kJ mol^−1^, making the proton transfer from N_2_H^+^ to acetone highly exothermic. Therefore, after protonation at the carbonyl group's oxygen, the vibrationally excited (CH_3_)_2_COH^+^ ion undergoes rearrangement to protonated propanal, CH_3_CH_2_CHOH^+^ [25, 27], which immediately eliminates C_2_H_4_ and forms the CH_2_OH^+^ fragment [28]. The isomerisation of (CH_3_)_2_COH^+^ to CH_3_CH_2_CHOH^+^ requires overcoming a transition state whose energy was estimated to be 226–268 kJ mol^−1^ above (CH_3_)_2_COH^+^ [29], which is not far from the above‐mentioned protonation exothermicity. Considering the experimental uncertainties of PA and activation energy values, as well as the fact that molecules and ions can be further energised in a discharge plasma, the suggested mechanism of the CH_2_OH^+^ ion does not seem unrealistic.

The formation of the third most abundant ion, C_3_H_3_^+^, observed under dry DBDI conditions is possible at elevated internal energies of acetone radical cations [30, 31], but it is likely to occur indirectly through fragmentation and rearrangement of the acetone radical cation [31] or via secondary reactions of ion and/or radical fragments. This conclusion is supported by the very low abundance of the *m/z* 39 product ion in the CID spectra of protonated acetone (Figure 3C and previously observed by McLafferty and Sakai [24]), indicating that this fragment does not originate from MH^+^ but could be formed through a different mechanism, most likely via secondary reactions.

A similar pattern was observed for other ketones. For 2‐heptanone under humid conditions, the dominant product ion was the protonated molecule at *m/z* 115, accompanied by its hydrated form at *m/z* 133 (Figure 3D). Under dry carrier gas conditions (Figure 3E), the spectrum underwent significant changes, showing extensive fragmentation. The most abundant fragment ions were *m/z* 55 and 45, together with less intense fragments at *m/z* 39, 41 and 43. The ion at *m/z* 32 and a fraction of the ion at *m/z* 29 could be the reagent ions. The CID spectra of protonated 2‐heptanone confirmed that ions at *m/z* 41, 45 and 55 and at least a fraction of ions at *m/z* 29 are generated from the fragmentation of the MH^+^ ion (see Figure 3F).

Although there is no doubt that the elemental composition of the ion *m/z* 45 is C_2_H_5_O^+^, most likely having a structure of protonated acetaldehyde, CH_3_CHOH^+^, the ion *m/z* 55 can be either C_4_H_7_^+^ or C_3_H_3_O^+^.

To determine whether the ion *m/z* 55 contains an oxygen atom, its CID spectrum was compared with the CID spectrum of the same nominal mass ion obtained by DBDI of 2‐heptene. Because these spectra were almost identical (see Figure S6B,C) and the ion *m/z* 55 from 2‐heptene should clearly be C_4_H_7_^+^, it can be concluded that the ion *m/z* 55 from 2‐heptanone has the same elemental composition. The most stable structure of the C_4_H_7_^+^ family is *anti*‐CH_3_CHCHCH_2_^+^ [32], and it may be speculated that it is formed from protonated 2‐heptanone by consecutive losses of H_2_O and propene molecules (note barely visible ions *m/z* 97 and 73 in Figure 3F). However, the real structural identity of the C_4_H_7_^+^ ion from DBDI of 2‐heptanone, as well as the detailed mechanism of its formation, remains unknown.

The mass spectrum of 2‐heptanone obtained under dry conditions differs significantly from that produced by electron ionisation [22]. Specifically, low abundances of 2‐heptanone's prominent EI fragments, *m/z* 58 and 43 [22], indicate that its molecular radical cation does not directly (i.e., via unimolecular fragmentation) contribute to the formation of ions observed in the dry DBDI spectrum.

Another investigated compound was 2‐pentanone, which showed very similar behaviour to previously described ketones. Under humid conditions, the dominant ions were the protonated molecule and its hydrated form. Under dry conditions, the dominant product was the fragment at *m/z* 45, which is also the main fragment observed in the CID spectra (see Figure S1).

### 3.3 Analysis of Carboxylic Acids Under Different Humidity Conditions

**FIGURE 4:**
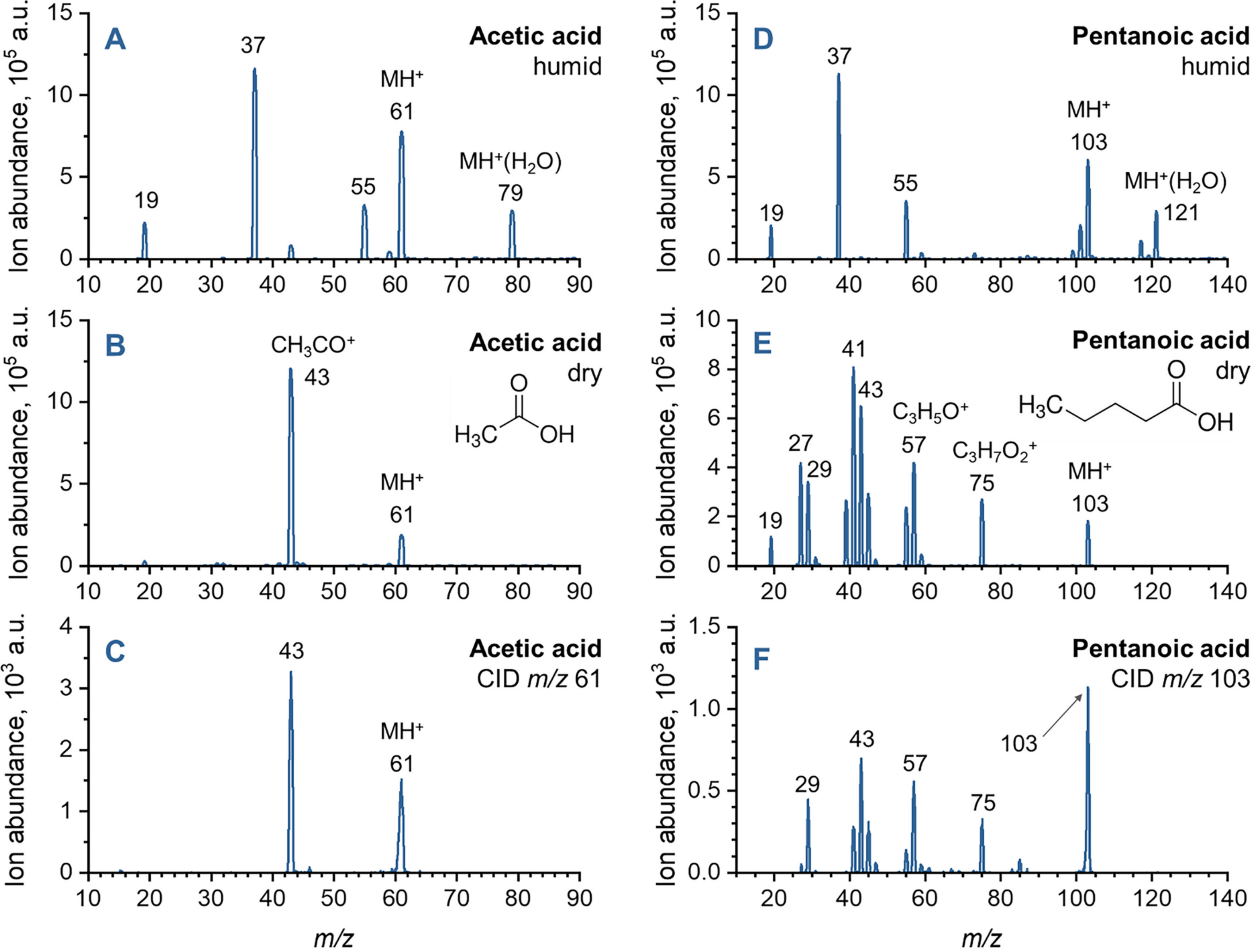
Mass spectra for acetic and pentanoic acids: (A, D) source DBDI spectra obtained under humidified carrier gas conditions, (B, E) source DBDI spectra obtained under dry carrier gas conditions and (C, F) CID spectra (Ar, 1.3 × 10^−3^ mbar, 20 V) of protonated molecules MH^+^ obtained under humid conditions.

Three short‐chain fatty acids were analysed—acetic acid, propanoic acid and pentanoic acid. In the humid DBDI mass spectra of all three acids (Figures 4A,D and S9A), peaks corresponding to protonated molecules, MH^+^, were observed. In addition, the hydrates of these species, that is, MH^+^(H_2_O) ions, were observed. For pentanoic acid, low‐abundant ions, which can be formally ascribed to dehydrogenation, that is, elimination of one or two dihydrogen molecules, were also observed (ions at *m/z* 119, 117, 101 and 99; see Figure 4A).

Unlike ketones, the short‐chain fatty acids are characterised by the presence of (M + H)^+^ ions in their dry DBDI mass spectra (Figures 4B,E and S9B). Besides these, abundant fragments resulting from the elimination of H_2_O are also present in the spectra of acetic and propanoic acids. The spectrum of pentanoic acid shows that the elimination of H_2_O is significantly suppressed. Instead, an elimination of a 28‐Da neutral fragment is present, leading to the ion *m/z* 75. The higher abundance of (M + H)^+^ ions observed for carboxylic acids under dry conditions could be related to the lower exothermicity of the proton‐transfer reactions compared with ketones. Because carboxylic acids have lower PAs, protonation by the proposed dry‐regime reagent ions (most plausibly N_2_H^+^) releases less excess energy, resulting in less internally excited protonated molecules and therefore reduced subsequent fragmentation. The CID mass spectrum of this ion (Figure S10B) is indistinguishable from that of protonated propanoic acid obtained under the humid DBDI conditions (Figure S10A). This clearly indicates that the 28‐Da neutral fragment is, in fact, an ethene molecule, C_2_H_4_.

The ions at *m/z* 57 from dry DBDI of propanoic and pentanoic acids are significantly different. Similarly to the elimination of H_2_O from acetic acid already mentioned in Section 3.2, the propanoic acid most likely yields acylium ions CH_3_CH_2_CO^+^, by elimination of a water molecule from MH^+^, whereas pentanoic acid yields butylium, C_4_H_9_^+^. This follows from comparing their CID mass spectra (Figure S11A,B) with that of *m/z* 57 from hexane (Figure S11C). The C_4_H_9_^+^ ion is characterised by the presence of ions *m/z* 41 and 39 (a loss of a molecule of CH_4_ with a subsequent loss of H_2_), whereas the CH_3_CH_2_CO^+^ ion forms an ethylium cation, C_2_H_5_^+^ (*m/z* 29), by losing CO, and the ketene radical cation, CH_2_CO^+•^ (*m/z* 42), by losing ^•^CH_3_. In the CID spectra of carboxylic acid, the higher abundance of (M + H)^+^ ions and less extensive fragmentation than those of ketones under comparable conditions were observed. This interpretation indicates that fragmentation pathways for protonated acids are energetically less favourable.

The CID mass spectra of ions at *m/z* 43 formed from propanoic and pentanoic acids under dry DBDI conditions (Figure S7C,D) are dramatically different from that of the acetyl cation (Figure S8A). They exhibit fragment ions at *m/z* 41, 39, 27 and 15 with slightly different abundances, suggesting the elemental composition of C_3_H_7_ because ions at *m/z* 39 and 27 cannot be fragments of an alternative acetyl cation, CH_3_CO^+^. The ion *m/z* 39 can only be C_3_H_3_^+^, formed from C_3_H_7_^+^ by losing four hydrogen atoms in total, and ion *m/z* 27, C_2_H_3_^+^, would need to originate from a loss of an oxygen atom if formed from CH_3_CO^+^, which is highly unlikely under the low‐energy multiple‐collision CID conditions used in this study. In contrast, the ion C_3_H_7_^+^ is known to lose a methane molecule [33].

To find a suitable precursor for the pure C_3_H_7_^+^ cation, regardless of structure, hexane and 2‐propanol were tested. The CID mass spectrum of the ion at *m/z* 43 from the dry DBDI of hexane is shown in Figure S8C and is surprisingly very similar to that of the acetyl cation, with only very low‐intensity additional peaks at *m/z* 42, 41, 39 and 27. In contrast, 2‐propanol produced an abundant ion at *m/z* 43 under dry DBDI conditions, with the CID spectrum shown in Figure S7A. Because it is known that the protonated 2‐propanol easily eliminates H_2_O and that the resulting 2‐propyl cation is the only primary ionic fragment [34], the spectrum in Figure S7A is considered as a spectrum of pure C_3_H_7_^+^. With this assumption, it can be determined that the ion at *m/z* 43 from propanoic acid consists of approximately 85% of C_3_H_7_^+^ and 15% of C_2_H_3_O^+^, whereas for pentanoic acid, it is approximately 70% of C_3_H_7_^+^ and 30% of C_2_H_3_O^+^.

Most of the fragment ions present in the dry DBDI mass spectra of the three acids studied (Figures 4B,E and S9B) are also present in the CID mass spectra of their MH^+^ ions from dry DBDI (Figures 4C,F and S9C), suggesting that protonated molecules play a key role in discharge chemistry under the dry conditions.

### 3.4 Differentiation of Isomers Under Dry Carrier Gas Conditions

Based on the formation of characteristic fragment ions observed under dry carrier gas conditions, structural isomers can be distinguished from one another. The reduced humidity in the ion source enhances fragmentation pathways that are specific to the molecular structure, allowing the identification of diagnostic ions unique to individual isomers. This approach provides valuable structural information that complements conventional soft ionisation spectra obtained under humid conditions.

From humid DBDI mass spectra, hexanone isomers (Figures S2A, S3A and S4A) cannot be distinguished, as only protonated molecules and their hydrates are formed. Therefore, CID MS is needed to distinguish them.

CID mass spectra of protonated molecules of the three isomeric hexanones are in Figures S2C, S3C and S4C. The spectra differ significantly from those published previously [35] because they were recorded under very different experimental conditions. However, the observed fragmentation patterns are qualitatively consistent with the literature and clearly reflect structural differences.

The CID spectrum of protonated 4‐methyl‐2‐pentanone (Figure S4C) is dominated by the peak at *m/z* 43. This ion most likely has an elemental composition of C_3_H_7_^+^ as can be judged from the CID spectrum of ion *m/z* 43 from dry DBDI of 4‐methyl‐2‐pentanone (*vide infra*) and reflects the presence of an isopropyl group in the molecule. On the other hand, the CID mass spectra of protonated 2‐hexanone (Figure S2C) and 3‐methyl‐2‐pentanone (Figure S3C) are dominated by peaks at *m/z* 45, most likely corresponding to protonated acetaldehyde, CH_3_CHOH^+^. The second most abundant fragment in the CID mass spectrum of protonated 2‐hexanone appears at *m/z* 55, whereas in protonated 3‐methyl‐2‐pentanone, the intense peak at *m/z* 59 is observed, enabling the unambiguous differentiation of these two species.

**FIGURE 5:**
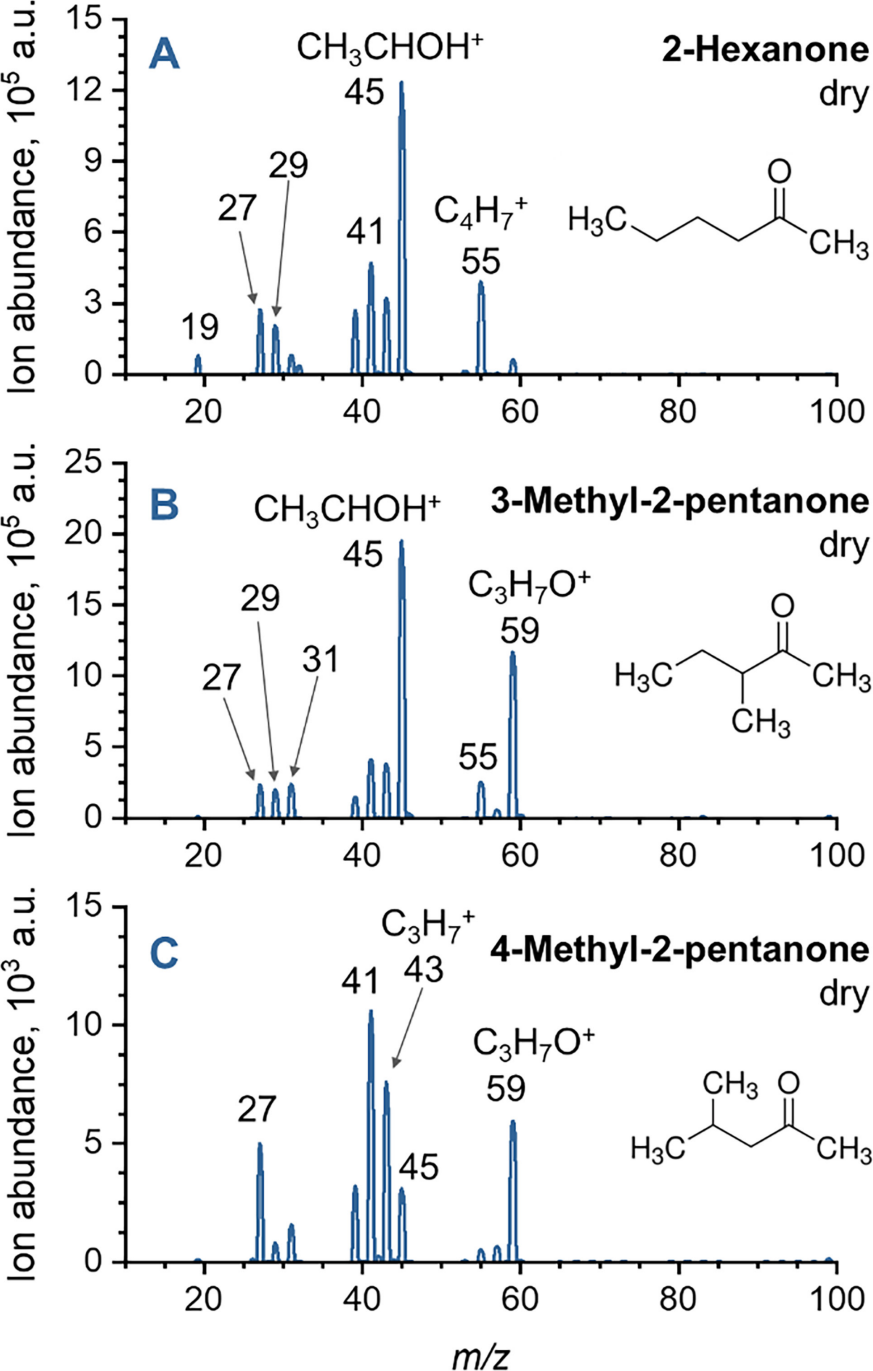
Dry DBDI mass spectra of (A) 2‐hexanone, (B) 3‐methyl‐2‐pentanone and (C) 4‐methyl‐2‐pentanone.

Using dry DBDI, isomer differentiation was possible based on source mass spectra (i.e., in Q1 mode) without the need for CID. Corresponding spectra are in Figure 5. Characteristic features of dry DBDI mass spectra of three isomeric hexanones can be summarised as follows.

The dry DBDI mass spectrum of 2‐hexanone (Figure 5A) is dominated by the ion at *m/z* 45, most likely with a structure of protonated acetaldehyde, which represents a global minimum on the singlet [C_2_, H_5_, O]^+^ potential energy surface [36]. This ion is formed by the elimination of a C_4_H_8_ molecule from the protonated 2‐hexanone, as the CID mass spectrum (Figure S2C) suggests.

Another characteristic ion was observed at *m/z* 55. Its CID mass spectrum (Figure S6A) almost perfectly matches the spectrum obtained from dry DBDI of 2‐heptene (Figure S6C), thus confirming its elemental composition as C_4_H_7_^+^.

Neither the protonated molecules (at *m/z* 101) nor the molecular radical cations (*m/z* 100) were observed in appreciable abundances, indicating that the above‐described fragmentation reactions are very efficient under the dry DBDI conditions.

There are two dominant peaks in the dry DBDI mass spectrum of 3‐methyl‐2‐pentanone at *m/z* 59 and 45 (Figure 5B). As in the case of 2‐hexene, the peak at *m/z* 45 can be attributed to the loss of C_4_H_8_ from protonated 3‐methyl‐2‐pentanone. The ion at *m/z* 59 likely has the elemental composition C_3_H_7_O^+^. This follows from a comparison of its CID mass spectrum (Figure S5A) with that of protonated acetone (Figure S5C), which are virtually identical. It also follows from this comparison that the elimination of a C_3_H_6_ molecule from protonated 3‐methyl‐2‐pentanone that yields C_3_H_7_O^+^ is accompanied by a molecular rearrangement, in which the methyl group at position 3 is transferred to position 2 or 4. An alternative two‐step elimination of C_3_H_6_ (e.g., to CH_4_ + C_2_H_2_) seems less likely, at least for thermochemical reasons.

The most abundant ion in the dry DBDI mass spectrum of 4‐methyl‐2‐pentanone (Figure 5C) was *m/z* 41 (most likely C_3_H_5_^+^), followed by ions at *m/z* 43, 59 and 27 in decreasing intensity. The ion at *m/z* 43 has a composition of C_3_H_7_^+^. This is clearly evident from its CID mass spectrum (Figure S7B), which is almost identical to that of the ion at *m/z* 43 from 2‐propanol (Figure S7A).

As in the case of 3‐methyl‐2‐pentanone, the CID mass spectrum of the ion at *m/z* 59 (Figure S5B) is indistinguishable from that of protonated acetone (Figure S5C) and markedly different from that of protonated propanal (Figure S5D). However, in the case of 4‐methyl‐2‐pentanone, the elimination of C_3_H_6_ does not require the transfer of a methyl group.

## 4 Conclusion

Humidity is a key factor affecting ionisation and downstream ion chemistry in active‐capillary DBDI. Under humidified carrier gas conditions, the spectra are dominated by proton transfer from hydronium and hydrated hydronium reagent ions, producing mainly protonated molecules MH^+^ along with their hydrates MH^+^(H_2_O)_n_ (and, at higher concentrations, proton‐bound complexes). Fragmentation was minimal, indicating a gentle chemical ionisation process. In this regard, humid DBDI is like SESI‐type ionisation, as the chemistry is driven by proton transfer from hydronium ions and their hydrates.

Switching to a dry carrier gas reduced the TIC and caused fragmentation. The primary fragment ions in dry DBDI spectra matched those in CID spectra of MH^+^ ions, indicating fragmentation from energetic protonated molecules rather than radical cations. This can be explained by exothermic dissociative proton transfer from the dry‐regime reagent ions at *m/z* 29, N_2_H^+^. It should be noted that the conclusion about the exothermic nature of protonation is also valid for possible HCO^+^ admixture.

These findings have practical analytical implications. Humid operation maximises signal intensity and favours stable MH^+^ formation, both of which are advantageous for robust detection. For quantitative use, humidity should therefore be controlled, because changes in water vapour concentration shift reagent ion distributions and can alter sensitivity. Dry operation reduces sensitivity but provides structure‐dependent fragment ions directly in the DBDI spectra, enabling differentiation of structural isomers without the need for CID. This suggests a useful selectivity–sensitivity trade‐off: humid DBDI for quantitative screening and dry DBDI for rapid structural discrimination, potentially combined with GC separation.

## Author Contributions

**Miroslav Polášek:** conceptualization, formal analysis, investigation, writing – original draft, writing – review and editing, methodology. **Patrik Španěl:** supervision, writing – review and editing. **Kseniya Dryahina:** conceptualization, formal analysis, visualization, project administration, writing – original draft, writing – review and editing, methodology, data curation.

## Conflicts of Interest

The authors declare no conflicts of interest.

## Supporting information


**Figure S1:** DBDI mass spectra for **2‐pentanone**: (A) under humidified carrier gas conditions, (B) under dry carrier gas conditions, and (C) CID mass spectra (Ar, 1.3 × 10^−3^ mbar, 20 V) of protonated molecules MH^+^ (*m/z* 87) obtained under humid conditions.
**Figure S2:** DBDI mass spectra for **2‐hexanone**: (A) under humidified carrier gas conditions, (B) under dry carrier gas conditions, and (C) CID mass spectra (Ar, 1.3 × 10^−3^ mbar, 20 V) of protonated molecules MH^+^ obtained under humid conditions.
**Figure S3:** DBDI mass spectra for **3‐methyl‐2‐pentanone**: (A) under humidified carrier gas conditions, (B) under dry carrier gas conditions, and (C) CID mass spectra (Ar, 1.3 × 10^−3^ mbar, 20 V) of protonated molecules MH^+^ obtained under humid conditions.
**Figure S4:** DBDI mass spectra for **4‐methyl‐2‐pentanone**: (A) under humidified carrier gas conditions, (B) under dry carrier gas conditions, and (C) CID mass spectra (Ar, 1.3 × 10^−3^ mbar, 20 V) of protonated molecules MH^+^ obtained under humid conditions.
**Figure S5:** CID mass spectra (Ar, 2.7 × 10^−3^ mbar, 20 V) of ions at ***m/z* 59** obtained under dry DBDI conditions from (A) **3‐methyl‐2‐pentanone** and (B) **4‐methyl‐2‐pentanone**, and as protonated molecules MH^+^ obtained under humid DBDI conditions for (C) **acetone** and (D) **propanal**.
**Figure S6:** CID mass spectra (Ar, 1.3 × 10^−3^ mbar, 20 V) of ions at ***m/z* 55** for (A) **2‐hexanone**, (B) **2‐heptanone**, and (C) **heptene** obtained under dry DBDI conditions.
**Figure S7:** CID mass spectra (Ar, 2.7 × 10^−3^ mbar, 20 V) of ions at ***m/z* 43** for (A) **2‐propanol**, (B) **4‐methyl‐2‐pentanone**, (C) **propanoic acid**, and (D) **pentanoic acid** obtained under dry DBDI conditions.
**Figure S8:** CID mass spectra (Ar, 2.7 × 10^−3^ mbar, 20 V) of ions at ***m/z* 43** for (A) **acetic acid**, (B) **acetone**, and (C) **hexane** obtained under dry DBDI conditions.
**Figure S9:** DBDI mass spectra for **propanoic acid**: (A) under humidified carrier gas conditions, (B) under dry carrier gas conditions, and (C) CID mass spectra (Ar, 1.3 × 10^−3^ mbar, 20 V) of protonated molecules MH^+^ obtained under humid conditions.
**Figure S10:** CID mass spectra (Ar, 2.7 × 10^−3^ mbar, 20 V) of ions at ***m/z* 75** obtained for (A) **propanoic acid** under humid DBDI conditions (i.e., MH^+^ ions) and (B) **pentanoic acid** under dry DBDI conditions. Note that the spectrum in Figure S9C was measured under a different collision gas pressure.
**Figure S11:** CID mass spectra (Ar, 2.7 × 10^−3^ mbar, 20 V) of ions at ***m/z* 57** obtained for (A) **propanoic acid**, (B) **pentanoic acid**, and (C) **hexane** under dry DBDI conditions.
**Figure S12:** Schematic diagram of the SICRIT ion source (Plasmion GmbH, Germany), an active‐capillary dielectric barrier discharge ionisation (DBDI) source with a ring‐to‐ring electrode configuration. The discharge is generated inside a dielectric capillary through which the sample and carrier gases flow.
**Figure S13:** (A) Time profiles of ion signals of *m/z* 29 reagent at dry conditions and *m/z* 45 product when CO_2_ is added at 1.5 min. (B) mass spectrum of filtered reagent ion *m/z* 29. (C) mass spectrum after addition of CO_2_.

## Data Availability

The data that support the findings of this study are available from the corresponding author upon reasonable request.
