# Supplementary material for: Effect of Humidity on Ionisation and Ion Chemistry in Active‐Capillary Dielectric Barrier Discharge Ionisation
Source: Rapid Commun Mass Spectrom. 2026 Jun 28;40(18):e70130. doi: 10.1002/rcm.70130 (PMC13311739; doi:10.1002/rcm.70130)
Supplement: Supplementary file 1 — Figure S1: DBDI mass spectra for 2‐pentanone: (A) under humidified carrier gas conditions, (B) under dry carrier gas conditions, and (C) CID mass spectra (Ar, 1.3 × 10^−3^ mbar, 20 V) of protonated molecules MH+ (m/z 87) obtained under humid conditions. Figure S2: DBDI mass spectra for 2‐hexanone: (A) under humidified carrier gas conditions, (B) under dry carrier gas conditions, and (C) CID mass spectra (Ar, 1.3 × 10^−3^ mbar, 20 V) of protonated molecules MH+ obtained under humid conditions. Figure S3: DBDI mass spectra for 3‐methyl‐2‐pentanone: (A) under humidified carrier gas conditions, (B) under dry carrier gas conditions, and (C) CID mass spectra (Ar, 1.3 × 10^−3^ mbar, 20 V) of protonated molecules MH+ obtained under humid conditions. Figure S4: DBDI mass spectra for 4‐methyl‐2‐pentanone: (A) under humidified carrier gas conditions, (B) under dry carrier gas conditions, and (C) CID mass spectra (Ar, 1.3 × 10^−3^ mbar, 20 V) of protonated molecules MH+ obtained under humid conditions. Figure S5: CID mass spectra (Ar, 2.7 × 10^−3^ mbar, 20 V) of ions at m/z 59 obtained under dry DBDI conditions from (A) 3‐methyl‐2‐pentanone and (B) 4‐methyl‐2‐pentanone, and as protonated molecules MH+ obtained under humid DBDI conditions for (C) acetone and (D) propanal. Figure S6: CID mass spectra (Ar, 1.3 × 10^−3^ mbar, 20 V) of ions at m/z 55 for (A) 2‐hexanone, (B) 2‐heptanone, and (C) heptene obtained under dry DBDI conditions. Figure S7: CID mass spectra (Ar, 2.7 × 10^−3^ mbar, 20 V) of ions at m/z 43 for (A) 2‐propanol, (B) 4‐methyl‐2‐pentanone, (C) propanoic acid, and (D) pentanoic acid obtained under dry DBDI conditions. Figure S8: CID mass spectra (Ar, 2.7 × 10^−3^ mbar, 20 V) of ions at m/z 43 for (A) acetic acid, (B) acetone, and (C) hexane obtained under dry DBDI conditions. Figure S9: DBDI mass spectra for propanoic acid: (A) under humidified carrier gas conditions, (B) under dry carrier gas conditions, and (C) CID mass spectra (Ar, 1.3 × 10^−3^ mbar, 20 V) of protonated molecules [file RCM-40-e70130-s001.docx]

Effect of humidity on ionisation and ion chemistry in active-capillary Dielectric Barrier Discharge Ionisation

Miroslav Polášek^1^, Patrik Španěl^1^, Kseniya Dryahina^1*^

Correspondence: Kseniya Dryahina [kseniya.dryahina@jh-inst.cas.cz](mailto:kseniya.dryahina@jh-inst.cas.cz)

# **Table of contents:**

**Figure S1.** DBDI mass spectra for **2-pentanone**: (A) under humidified carrier gas conditions, (B) under dry carrier gas conditions, and (C) CID mass spectra (Ar, 1.3×10^-3^ mbar, 20 V) of protonated molecules MH^+^ (*m/z* 87) obtained under humid conditions.

**Figure S2.** DBDI mass spectra for **2-hexanone**: (A) under humidified carrier gas conditions, (B) under dry carrier gas conditions, and (C) CID mass spectra (Ar, 1.3×10^-3^ mbar, 20 V) of protonated molecules MH^+^ obtained under humid conditions.

**Figure S3.** DBDI mass spectra for **3-methyl-2-pentanone**: (A) under humidified carrier gas conditions, (B) under dry carrier gas conditions, and (C) CID mass spectra (Ar, 1.3×10^-3^ mbar, 20 V) of protonated molecules MH^+^ obtained under humid conditions.

**Figure S4.** DBDI mass spectra for **4-methyl-2-pentanone**: (A) under humidified carrier gas conditions, (B) under dry carrier gas conditions, and (C) CID mass spectra (Ar, 1.3×10^-3^ mbar, 20 V) of protonated molecules MH^+^ obtained under humid conditions.

**Figure S5.** CID mass spectra (Ar, 2.7×10^-3^ mbar, 20 V) of ions at ***m/z* 59** obtained under dry DBDI conditions from (A) **3-methyl-2-pentanone** and (B) **4-methyl-2-pentanone**, and as protonated molecules MH^+^ obtained under humid DBDI conditions for (C) **acetone** and (D) **propanal**.

**Figure S6.** CID mass spectra (Ar, 1.3×10^-3^ mbar, 20 V) of ions at ***m/z* 55** for (A) **2-hexanone**, (B) **2-heptanone**, and (C) **heptene** obtained under dry DBDI conditions.

**Figure S7.** CID mass spectra (Ar, 2.7×10^-3^ mbar, 20 V) of ions at ***m/z* 43** for (A) **2-propanol**, (B) **4-methyl-2-pentanone**, (C) **propanoic acid**, and (D) **pentanoic acid** obtained under dry DBDI conditions.

**Figure S8.** CID mass spectra (Ar, 2.7×10^-3^ mbar, 20 V) of ions at ***m/z* 43** for (A) **acetic acid**, (B) **acetone**, and (C) **hexane** obtained under dry DBDI conditions.

**Figure S9.** DBDI mass spectra for **propanoic acid**: (A) under humidified carrier gas conditions, (B) under dry carrier gas conditions, and (C) CID mass spectra (Ar, 1.3×10^-3^ mbar, 20 V) of protonated molecules MH^+^ obtained under humid conditions.

**Figure S10.** CID mass spectra (Ar, 2.7×10^-3^ mbar, 20 V) of ions at ***m/z* 75** obtained for (A) **propanoic acid** under humid DBDI conditions (i.e., MH^+^ ions) and (B) **pentanoic acid** under dry DBDI conditions. Note that the spectrum in Figure S9C was measured under a different collision gas pressure.

**Figure S11.** CID mass spectra (Ar, 2.7×10^-3^ mbar, 20 V) of ions at ***m/z* 57** obtained for (A) **propanoic acid**, (B) **pentanoic acid**, and (C) **hexane** under dry DBDI conditions.

**Figure S12.** Schematic diagram of the SICRIT^®^ ion source (Plasmion GmbH, Germany), an active-capillary dielectric barrier discharge ionisation (DBDI) source with a ring-to-ring electrode configuration. The discharge is generated inside a dielectric capillary through which the sample and carrier gases flow.

**Figure S13.** (A) Time profiles of ion signals of *m/z* 29 reagent at dry conditions and *m/z* 45 product when CO_2_ is added at 1.5 minutes. (B) mass spectrum of filtered reagent ion *m/z* 29. (C) mass spectrum after addition of CO_2_.


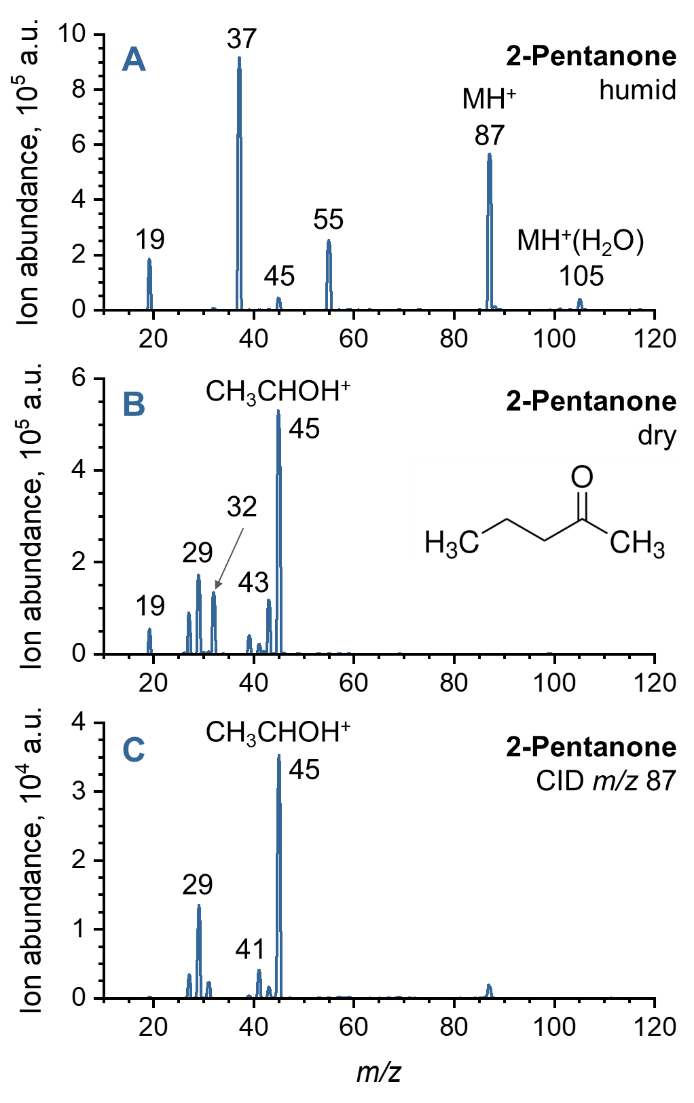


**Figure S1.** DBDI mass spectra for 2-pentanone: (A) under humidified carrier gas conditions, (B) under dry carrier gas conditions, and (C) CID mass spectra (Ar, 1.3×10^-3^ mbar, 20 V) of protonated molecules MH^+^ (*m/z* 87) obtained under humid conditions.


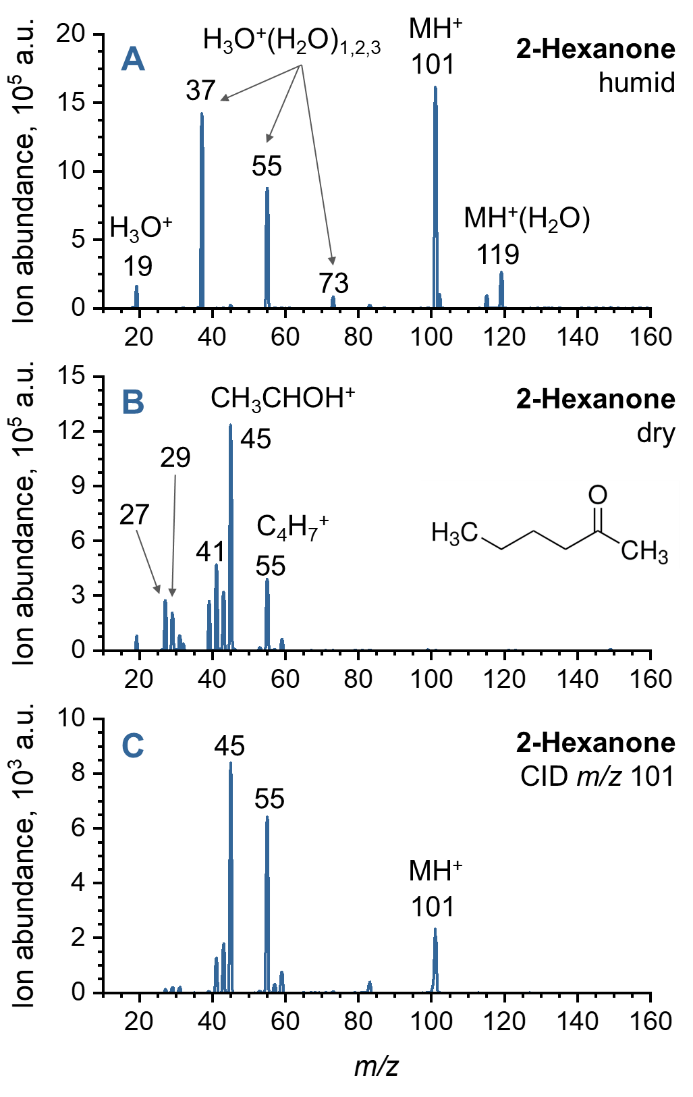


**Figure S2.** DBDI mass spectra for 2-hexanone: (A) under humidified carrier gas conditions, (B) under dry carrier gas conditions, and (C) CID mass spectra (Ar, 1.3×10^-3^ mbar, 20 V) of protonated molecules MH^+^ obtained under humid conditions.


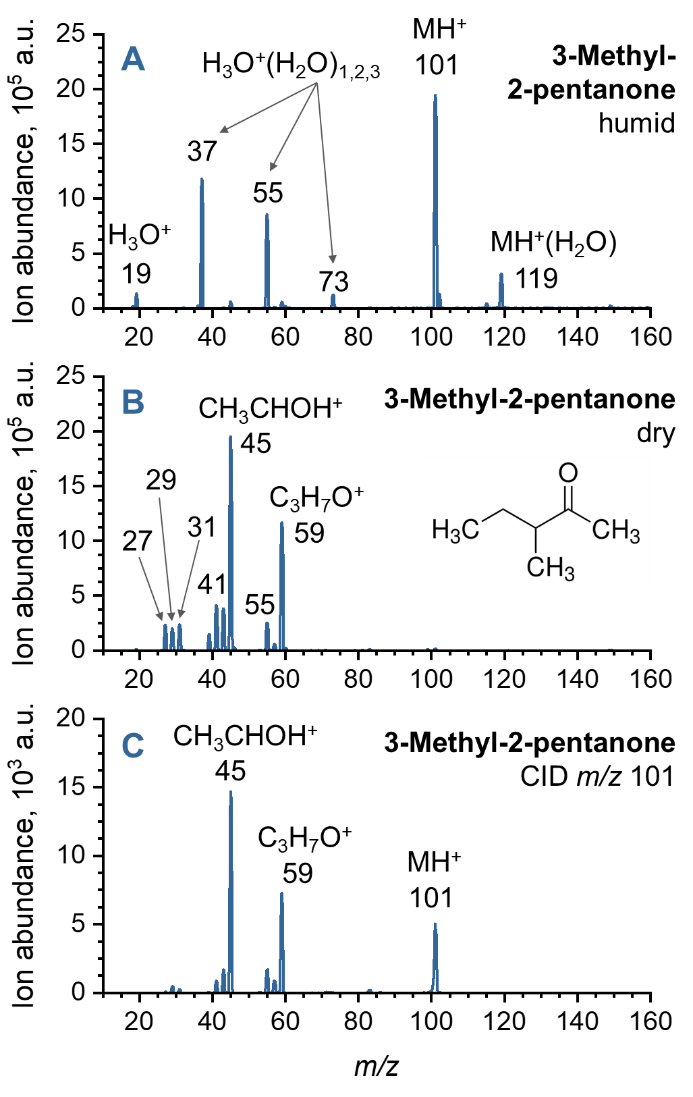


**Figure S3.** DBDI mass spectra for 3-methyl-2-pentanone: (A) under humidified carrier gas conditions, (B) under dry carrier gas conditions, and (C) CID mass spectra (Ar, 1.3×10^-3^ mbar, 20 V) of protonated molecules MH^+^ obtained under humid conditions.


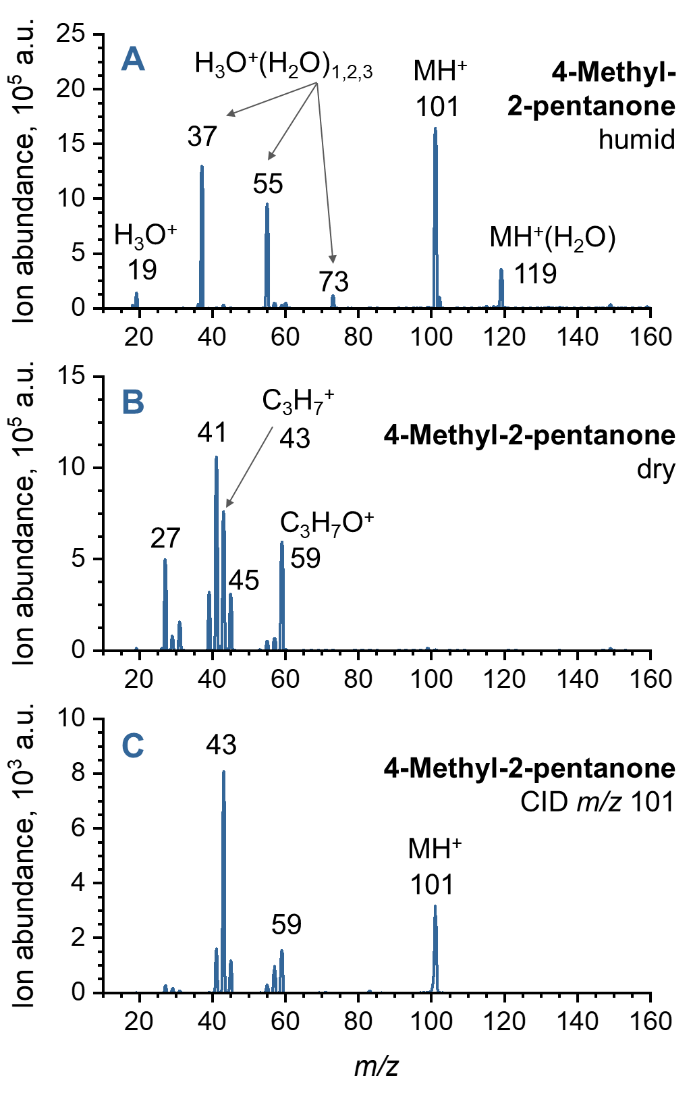


**Figure S4.** DBDI mass spectra for 4-methyl-2-pentanone: (A) under humidified carrier gas conditions, (B) under dry carrier gas conditions, and (C) CID mass spectra (Ar, 1.3×10^-3^ mbar, 20 V) of protonated molecules MH^+^ obtained under humid conditions.


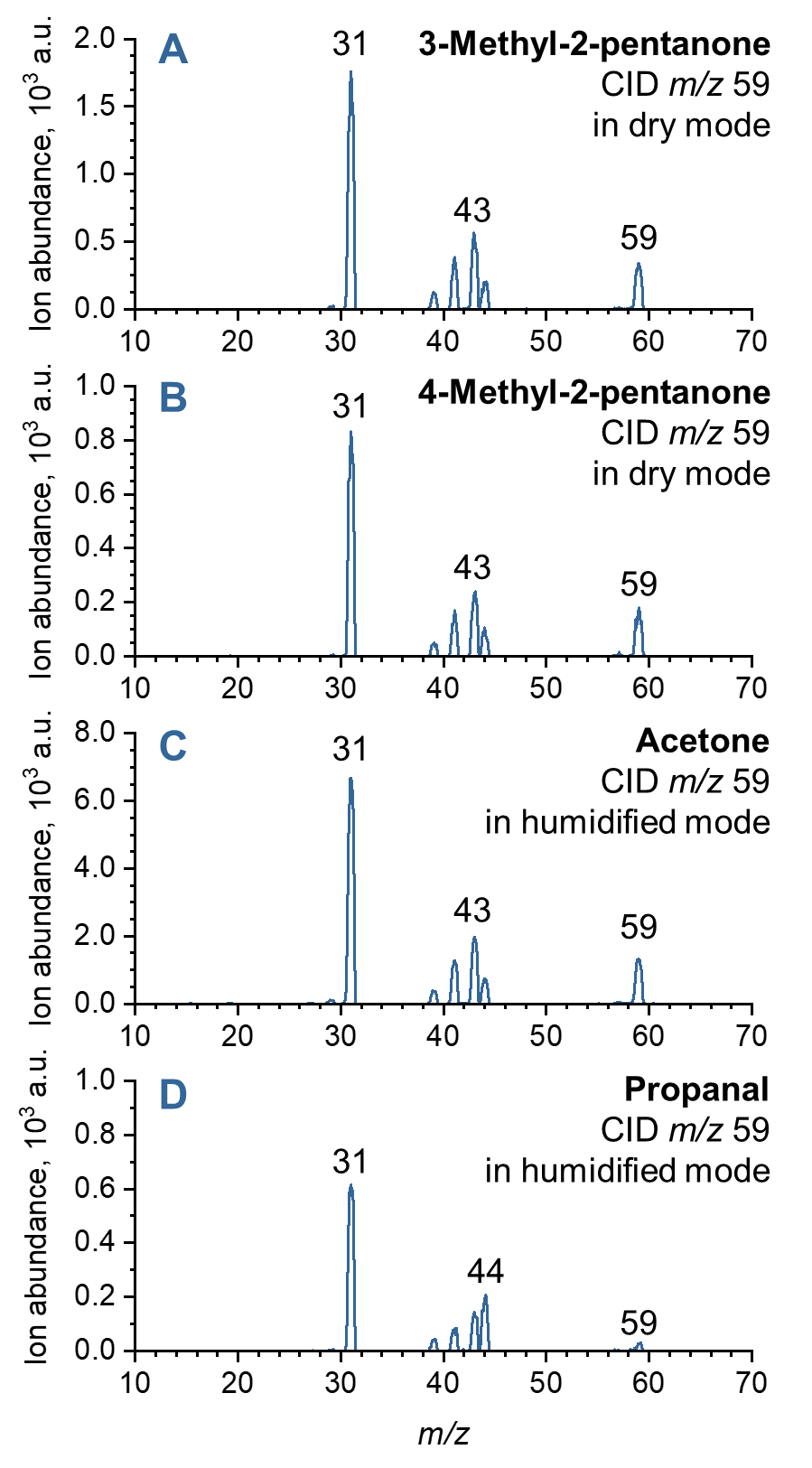


**Figure S5.** CID mass spectra (Ar, 2.7×10^-3^ mbar, 20 V) of ions at *m/z* 59 obtained under dry DBDI conditions from (A) 3-methyl-2-pentanone and (B) 4-methyl-2-pentanone, and as protonated molecules MH^+^ obtained under humid DBDI conditions for (C) acetone and (D) propanal.


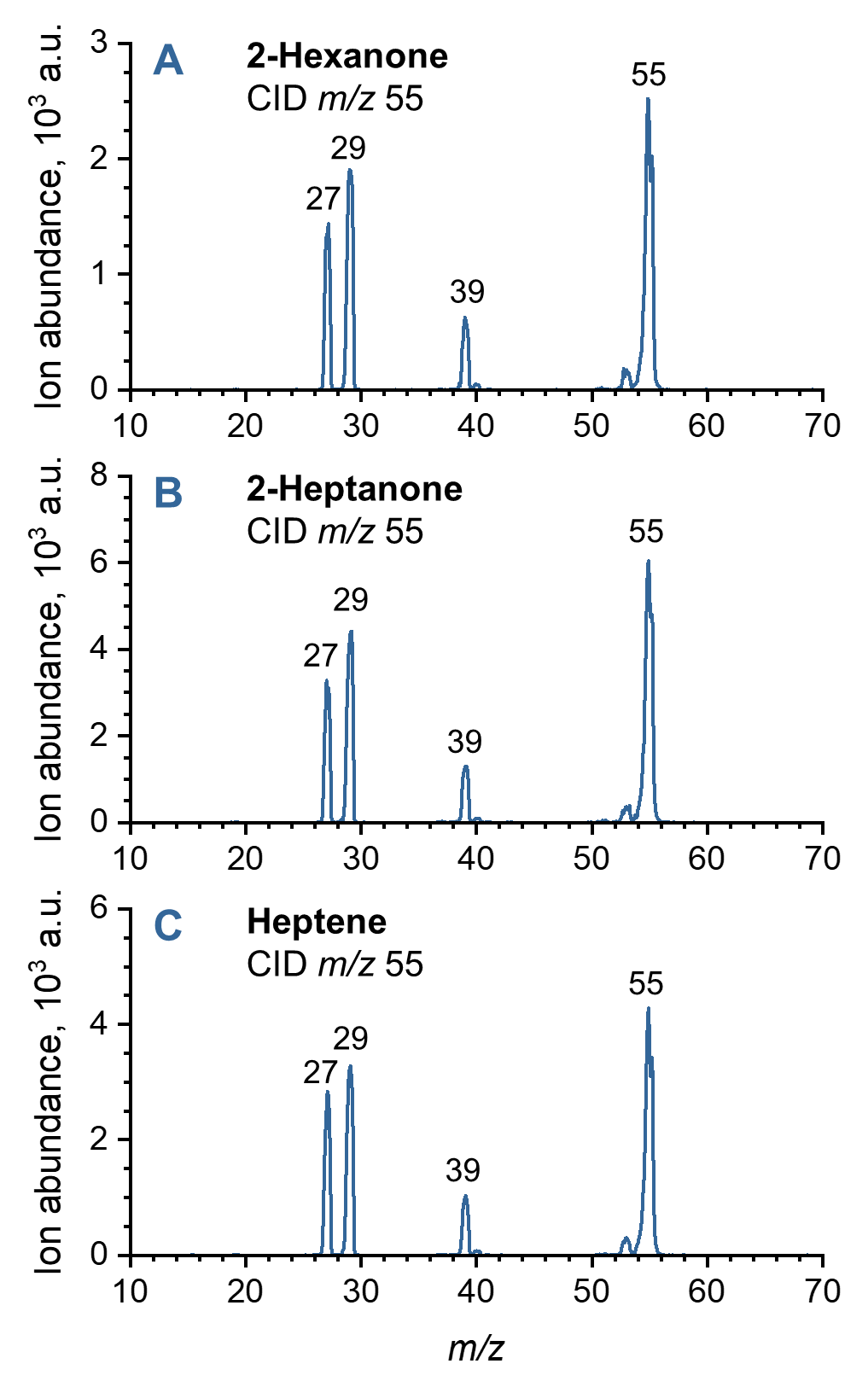


**Figure S6.** CID mass spectra (Ar, 1.3×10^-3^ mbar, 20 V) of ions at *m/z* 55 for (A) 2-hexanone,
(B) 2-heptanone, and (C) heptene obtained under dry DBDI conditions.


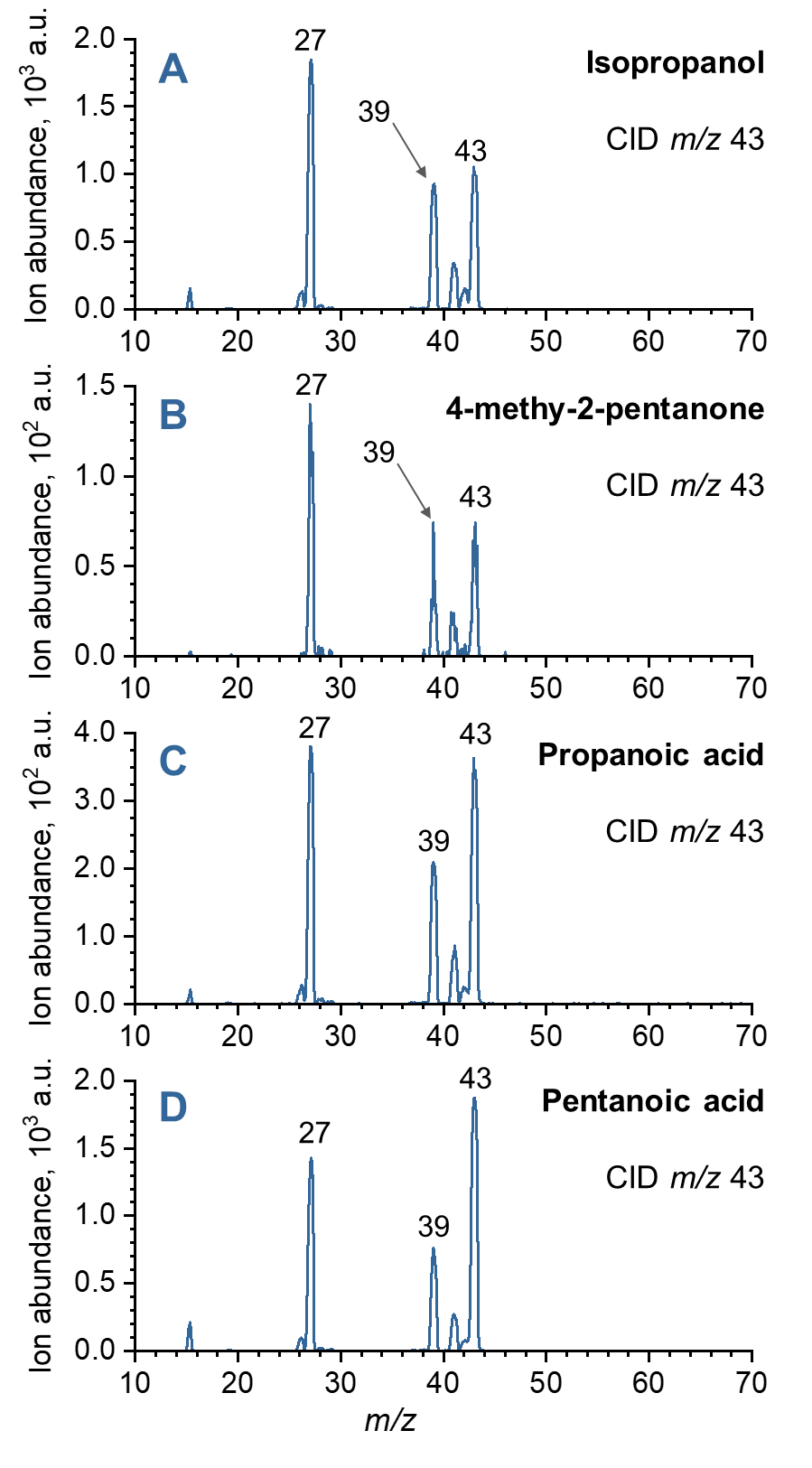


**Figure S7.** CID mass spectra (Ar, 2.7×10^-3^ mbar, 20 V) of ions at *m/z* 43 for (A) 2-propanol,
(B) 4-methyl-2-pentanone, (C) propanoic acid, and (D) pentanoic acid obtained under dry DBDI conditions.


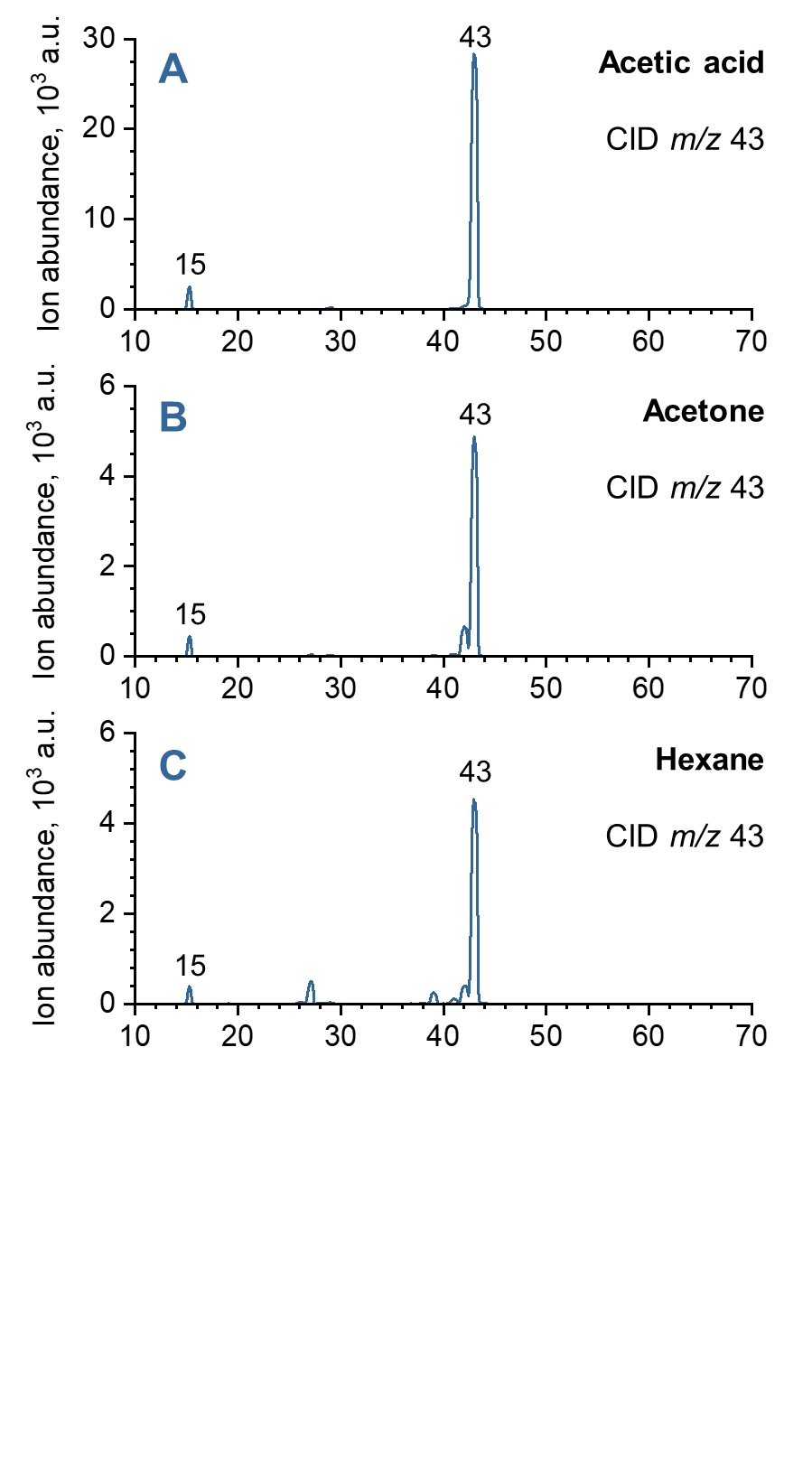


**Figure S8.** CID mass spectra (Ar, 2.7×10^-3^ mbar, 20 V) of ions at *m/z* 43 for (A) acetic acid,
(B) acetone, and (C) hexane obtained under dry DBDI conditions.


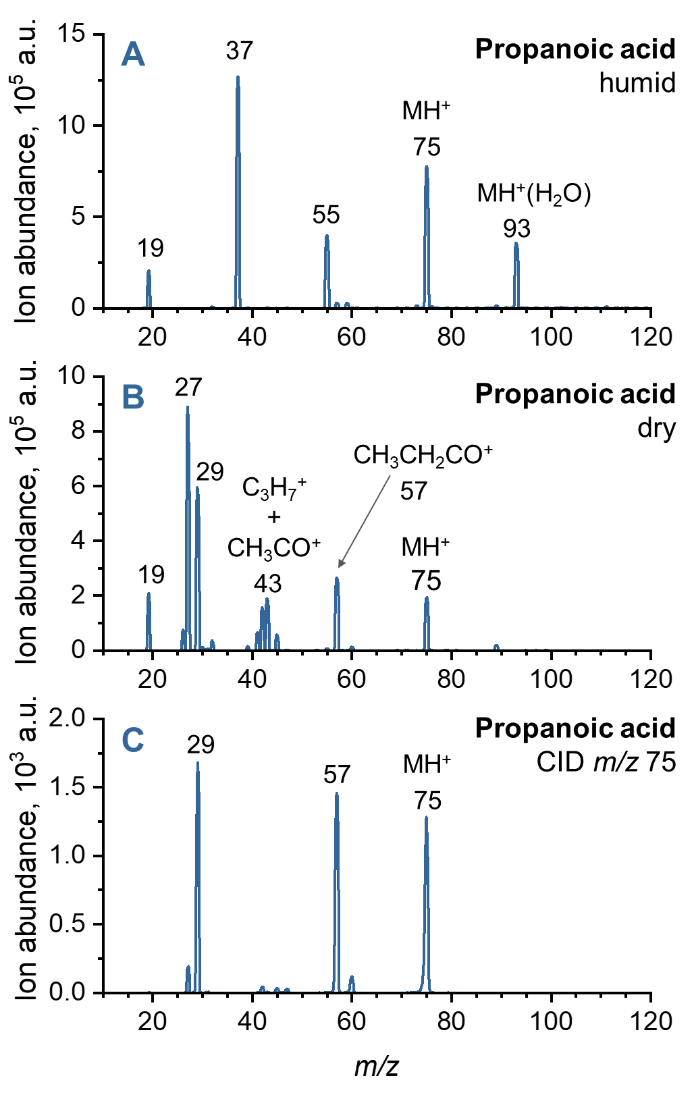


**Figure S9.** DBDI mass spectra for propanoic acid: (A) under humidified carrier gas conditions, (B) under dry carrier gas conditions, and (C) CID mass spectra (Ar, 1.3×10^-3^ mbar, 20 V) of protonated molecules MH^+^ obtained under humid conditions.


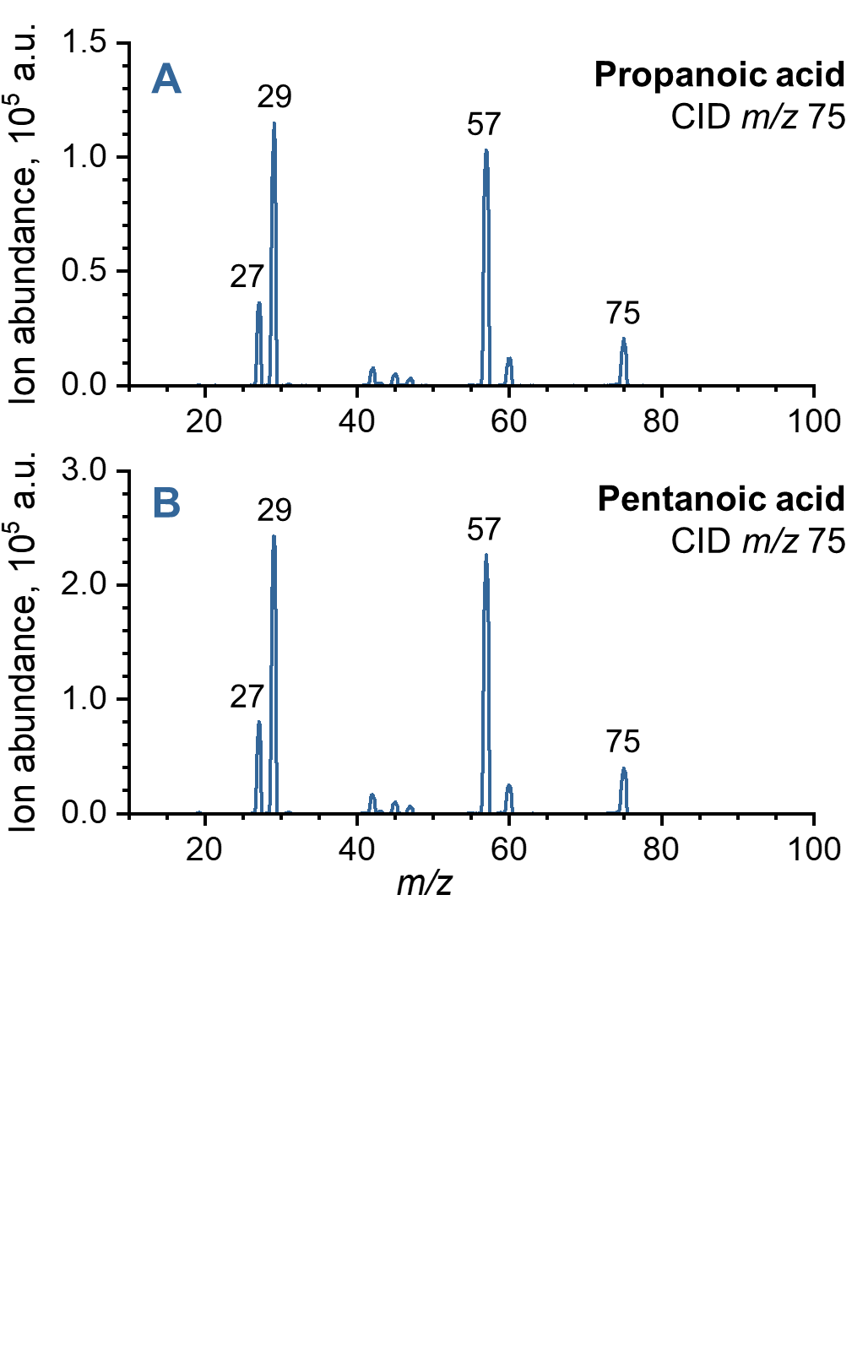


**Figure S10.** CID mass spectra (Ar, 2.7×10^-3^ mbar, 20 V) of ions at *m/z* 75 obtained for (A) propanoic acid under humid DBDI conditions (i.e., MH^+^ ions) and (B) pentanoic acid under dry DBDI conditions. Note that the spectrum in Figure S9C was measured under a different collision gas pressure.


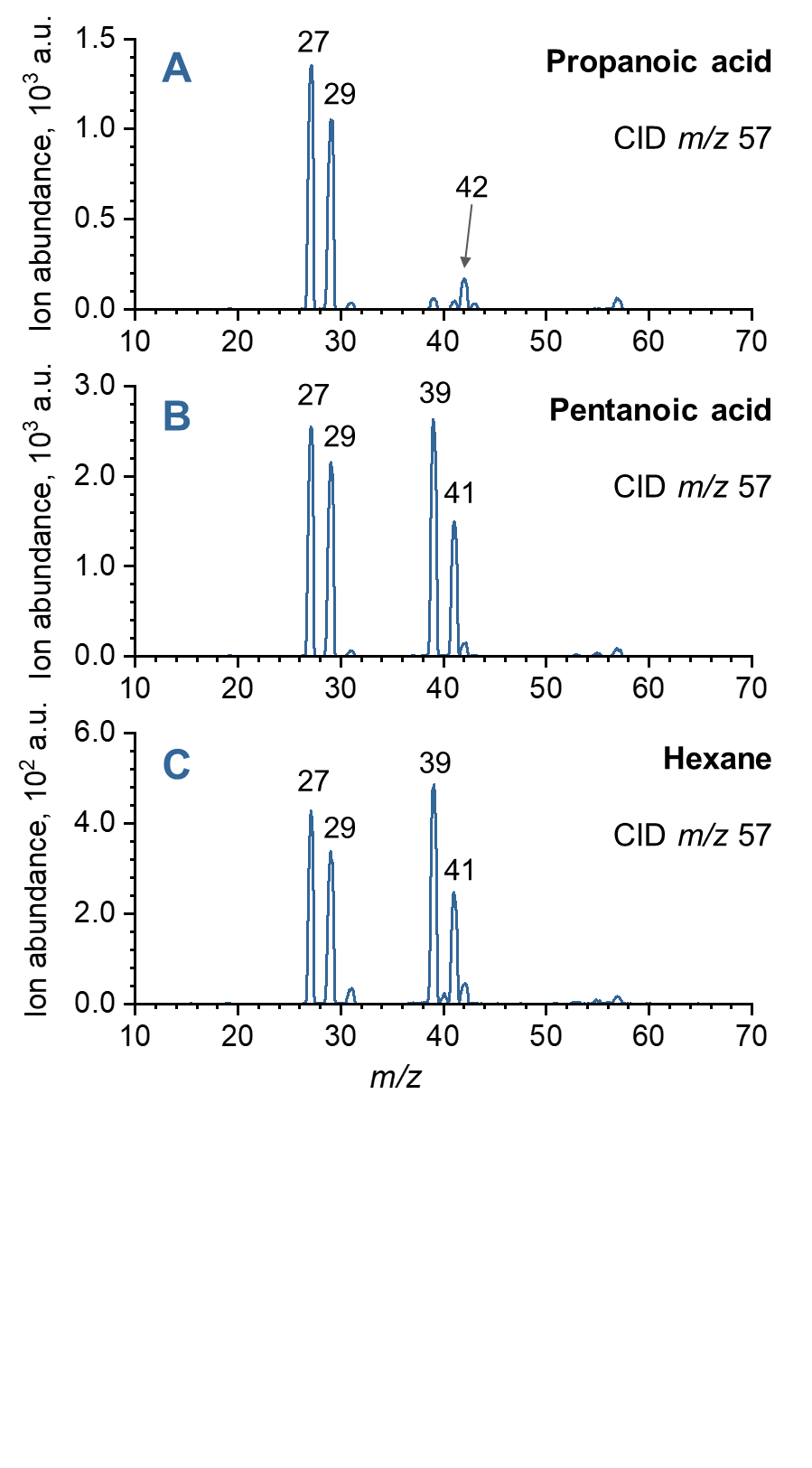


**Figure S11.** CID mass spectra (Ar, 2.7×10^-3^ mbar, 20 V) of ions at *m/z* 57 obtained for
(A) propanoic acid, (B) pentanoic acid, and (C) hexane under dry DBDI conditions.

**
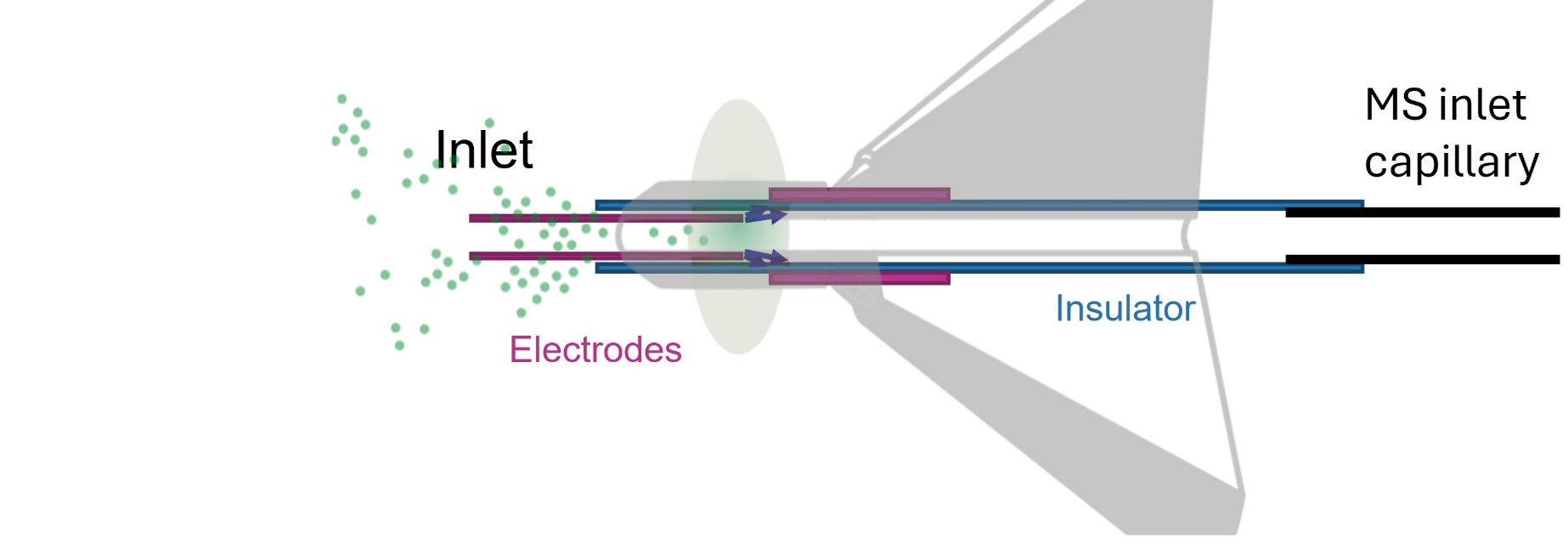
**

**Figure S12.** Schematic diagram of the SICRIT^®^ ion source (Plasmion GmbH, Germany), an active-capillary dielectric barrier discharge ionisation (DBDI) source with a ring-to-ring electrode configuration. The discharge is generated inside a dielectric capillary through which the sample and carrier gases flow.

**

**

**Figure S13.** (A) Time profiles of ion signals of *m/z* 29 reagent at dry conditions and *m/z* 45 product when CO_2_ is added at 1.5 minutes. (B) mass spectrum of filtered reagent ion *m/z* 29. (C) mass spectrum after addition of CO_2_.
